# Acetaldehyde as a drug of abuse: insight into AM281 administration on operant-conflict paradigm in rats

**DOI:** 10.3389/fnbeh.2013.00064

**Published:** 2013-06-11

**Authors:** Fulvio Plescia, Anna Brancato, Rosa A. M. Marino, Carla Cannizzaro

**Affiliations:** Laboratory of Neuropsychopharmacology, Section of Pharmacology, Department of Sciences for Health Promotion and Mother and Child Care “Giuseppe D'Alessandro”, University of PalermoPalermo, Italy

**Keywords:** acetaldehyde, lever pressing, relapse, Geiller–Seifter procedure, CB1 receptor blockade/antagonism

## Abstract

Increasing evidence focuses on acetaldehyde (ACD) as the mediator of the rewarding and motivational properties of ethanol. Indeed, ACD stimulates dopamine release in the nucleus accumbens and it is self-administered under different conditions. Besides the dopaminergic transmission, the endocannabinoid system has been reported to play an important role in ethanol central effects, modulating primary alcohol rewarding effect, drug-seeking, and relapse behavior. Drug motivational properties are highlighted in operant paradigms which include response-contingent punishment, a behavioral equivalent of compulsive drug use despite adverse consequences. The aim of this study was thus to characterize ACD motivational and rewarding properties employing an operant-conflict paradigm in which rats, trained to lever press in order to get ACD solution (0.9%), undergo extinction, reinstatement and conflict sessions, according to a modified Geller–Seifter procedure. Furthermore, the role played by CB1 receptor system in modulating ACD-induced effects were investigated through the administration of CB1 receptor antagonist, AM281 (1 mg/kg, i.p.) during the extinction-, relapse-, and conflict-experiments. Our results indicate that ACD is able to induce and maintain an operant behavior, a high number of responses during extinction, an increase in the lever presses during the reinstatement phase, and a higher emission of punished responses during the conflict experiments, when compared to controls. The administration of AM281 is able to decrease ACD-seeking behavior during extinction, the number of lever presses during reinstatement and to strongly decrease the punished responses for ACD. Our data strengthen the idea that ACD may be responsible for the central effects of ethanol, and pinpoint at the CB1 system as one of the neural substrates underlying its addictive properties.

## Introduction

Evidence obtained in preclinical studies suggests that Acetaldehyde (ACD), the first metabolite of ethanol, is biologically active and may play a pivotal role in the rewarding, motivational and addictive properties of alcohol, as recently reviewed by Correa et al. (2012) and Deehan et al. (2013a).

ACD is obtained from ethanol oxidative metabolism, which occurs by peripheral alcohol dehydrogenase, and by central catalase and CYP2E1 (Zimatkin et al., 1998; Arizzi-LaFrance et al., 2006; Zakhari, 2006; Jamal et al., 2007). High blood levels of ACD enter the brain, likely overwhelming the aldehyde dehydrogenase present in the blood-brain barrier (Quertemont et al., 2005).

ACD is able to affect dopamine neurotransmission, increasing neuronal firing in the ventral tegmental area (VTA), thus stimulating DA release in the nucleus accumbens (NAcc) shell (Melis et al., 2007; Deehan et al., 2013b). Recent reports show that the intra-VTA administration of a lentiviral vector, able to inhibit catalase synthesis, and hence central ACD production, nearly abolishes voluntary ethanol intake, as well as decreases ethanol-induced DA release in the NAcc shell (Karahanian et al., 2011), further supporting the compelling theory that ethanol may be acting as a prodrug. Behavioral studies confirm that ACD administration is able to induce conditioned place preference (Smith et al., 1984; Peana et al., 2008); furthermore rats readily self-administer ACD solution in operant conditions through several routes: centrally (Amit et al., 1977; Brown et al., 1979; Rodd et al., 2005), and peripherally (Myers et al., 1982; Peana et al., 2010; Cacace et al., 2012).

Besides the dopaminergic transmission, the brain endocannabinoid (EC) system plays an important role in value-attribution processing and in the modulation of reward-seeking behavior for different drugs of abuse (Serrano and Parsons, 2011), in view of its role as fine modulator of incoming inputs within the VTA (Melis et al., 2012).

In particular, CB1 receptor manipulation is reported to affect ethanol-related behavior, and in fact CB1 antagonism decreases both voluntary ethanol intake and relapse to ethanol in several experimental models (Arnone et al., 1997; Colombo et al., 1998; Gallate and McGregor, 1999; Cippitelli et al., 2005; Economidou et al., 2006; Femenía et al., 2010; De Bruin et al., 2011; Getachew et al., 2011), suggesting that CB1 receptor blockade reduces the rewarding value of ethanol. In turn, chronic administration of ethanol is associated with increased concentrations of endocannabinoids, in accordance with a reduction in the activity of their removal mechanisms, and in CB1 receptor expression, thus affecting the system as a whole (Basavarajappa et al., 1998, 2000, 2003; Vinod et al., 2006).

Given these premises, it is worth focusing on CB1 receptor involvement in ACD self-administration, employing an operant conditioning paradigm which may reliably model the distinct phases of the addiction cycle. Indeed, punishment resistance, which represents the behavioral equivalent of compulsive drug use despite negative consequences (Deroche-Gamonet et al., 2004), is considered as a mandatory component in mirroring the addictive phenotype, besides drug taking, drug seeking and relapse (Marchant et al., 2013). Hence, by the assessment of the capacity of orally self-administered ACD to induced and maintain an operant behavior after forced abstinence, and in the presence of an aversive stimulus, according to a programmed schedule of responding (Cannizzaro et al., 2011; Cacace et al., 2012), the evaluation of the influence of CB1 receptor blockade by AM281 was carried out on ACD-seeking and relapse, and on compulsive-like behavior.

## Materials and methods

### Animals

Adult male Wistar rats (Harlan, Udine, Italy) weighing 250 to 300 g, were housed two per cage and maintained on a 12-h light/dark cycle, under controlled environmental conditions (temperature 22 ± 2°C; humidity 55 ± 10%) with food and water available *ad-libitum*. During operant behavior experiments they were water-restricted and allowed to drink only 1 h/day at the end of the experimental sessions. Water intake was recorded. All subjects were experimentally naive and randomly assigned to the following groups (*n* = 16): ACD-drinking rats (ACD) which self-administered a solution of ACD (0.9% v/v) and water-drinking rats (CTR) which self-administered water. All experiments were in accordance with the Committee for Use of Experimental Animals of the University of Palermo, the Italian legislation D.L. 116/1992 and the European Union Council Directive 2010/63, dealing with research on experimental animals.

### Drugs

ACD 99.98% (Sigma-Aldrich, Milan, Italy) was diluted in tap water, in order to achieve a final concentration of 0.9% v/v (0.450 ml ACD in 50 ml of water). ACD solution was daily prepared, sealed with Parafilm (American Can Company), stored at 4°C during experiments, aiming at avoiding concentration loss (Cacace et al., 2012).

The CB1 selective cannabinoid antagonist AM281 (Sigma-Aldrich, Milan, Italy) was dissolved in a vehicle of Tween 80 (3%) in saline solution (0.9% NaCl), and administered intraperitoneally (1 mg/kg).

### Apparatus

The experimental sessions were carried out in a custom-built operant-conditioning chamber (30 × 28 × 37 cm), included in a dim-illuminated, ventilated, sound-attenuating cubicle. The chamber was equipped with one active lever and a cup that collected liquid from a corked reservoir, aiming at ACD solution preservation from evaporation, with a solenoid-actuated delivery system. It assured the delivery of 0.05 ml of solution for each lever pressing. Moreover, a foot-shock generator was able to deliver a constant-current, intermittent, inescapable, foot-shock (0.2 mA) to the chamber grid floor. A light-cue above the lever turned on during the punished period, allowing the animals to be aware of the aversive stimulus. Animal performance was recorded on a counter connected to the chamber. The devices were thoroughly cleaned before the introduction of each animal to ensure that the particular rat's behavior was not affected by the detection of another rat's scent.

### Operant self-administration procedure

The lever pressing procedure was scheduled into four different periods: Training—rewarded responses under a continuous schedule of reinforcement; Extinction—non rewarded responses; Relapse—reinstatement of the reinforced operant behavior following 1 week-deprivation; Conflict—rewarded responses cyclically associated with a 0.2 mA footshock.

#### Shaping and training

Animals underwent water deprivation for 23 h and then they were shaped to lever press in order to obtain water on a continuous reinforcement schedule (fixed ratio 1), until they achieved a steady performance. Afterwards, during the *Training* session rats orally self-administered ACD solution or water, according to their group, in the operant chamber, under a fixed ratio 1, along 21 days. For each operant response, the system delivered 0.05 ml of water or 0.90% v/v ACD solution. The number of lever presses was automatically recorded. Animals were tested each day at the same time (9:00 to 14:00), and each trial lasted 20 min.

#### Extinction

Animals underwent an operant responding session during which reward delivery was suspended. The number of lever presses at the end of the 20 min session was recorded for both ACD and Control groups.

#### Deprivation

ACD self-administration was suspended for 7 days to achieve a forced abstinence. Rats were left undisturbed in their home cages and received water and food *ad-libitum*.

#### Relapse

After the deprivation period, rats were exposed again to lever pressing in the operant chamber with a fixed ratio 1 response schedule for 7 days. Responses for ACD or water were recorded during the 20 min-experimental sessions.

#### Conflict

This protocol represented a modification of the Geller–Seifter paradigm, a procedure in which a positive reinforcement (water or ACD solution) is earned by an operant response; however, delivery of the positive reinforce is paired with an aversive stimulus, as a footshock. In our study, animals underwent alternatively unpunished and punished responses according to “3 minutes–1 minute” schedule. The session started and ended with an unpunished interval. The punishment was signaled by a cue-light triggered by the response. During the punished response interval, lever presses were rewarded with the solution and coupled with a mild footshock of 0.2 mA. The number of unpunished and punished responses was automatically recorded.

#### Treatment

CB1 antagonist AM281 was administered to ACD-group 30 min before the experimental sessions, during extinction (1 day), relapse (7 days), and conflict (7 days). Control rats were administered intraperitoneally with vehicle.

#### Statistical analysis

***Operant-drinking behavior***. A Two-Way analysis of variance (ANOVA) was conducted on the number of lever presses during the training, extinction-, relapse- and conflict-sessions, as dependent variables, with “ACD self-administration” (treatment) as between-subjects factor, and “days” as repeated measurement factor. When necessary, simple main effects and *post-hoc* comparisons were calculated with Bonferroni post-test (α = 0.05). Differences were considered statistically significant if *p* < 0.05. Furthermore, to compare the effect of AM281 treatment on ACD-seeking behavior during extinction, a 2-tailed Student's *t*-test for unpaired measures was employed.

## Results

### Operant self-administration

#### Training period

**Figure 1:**
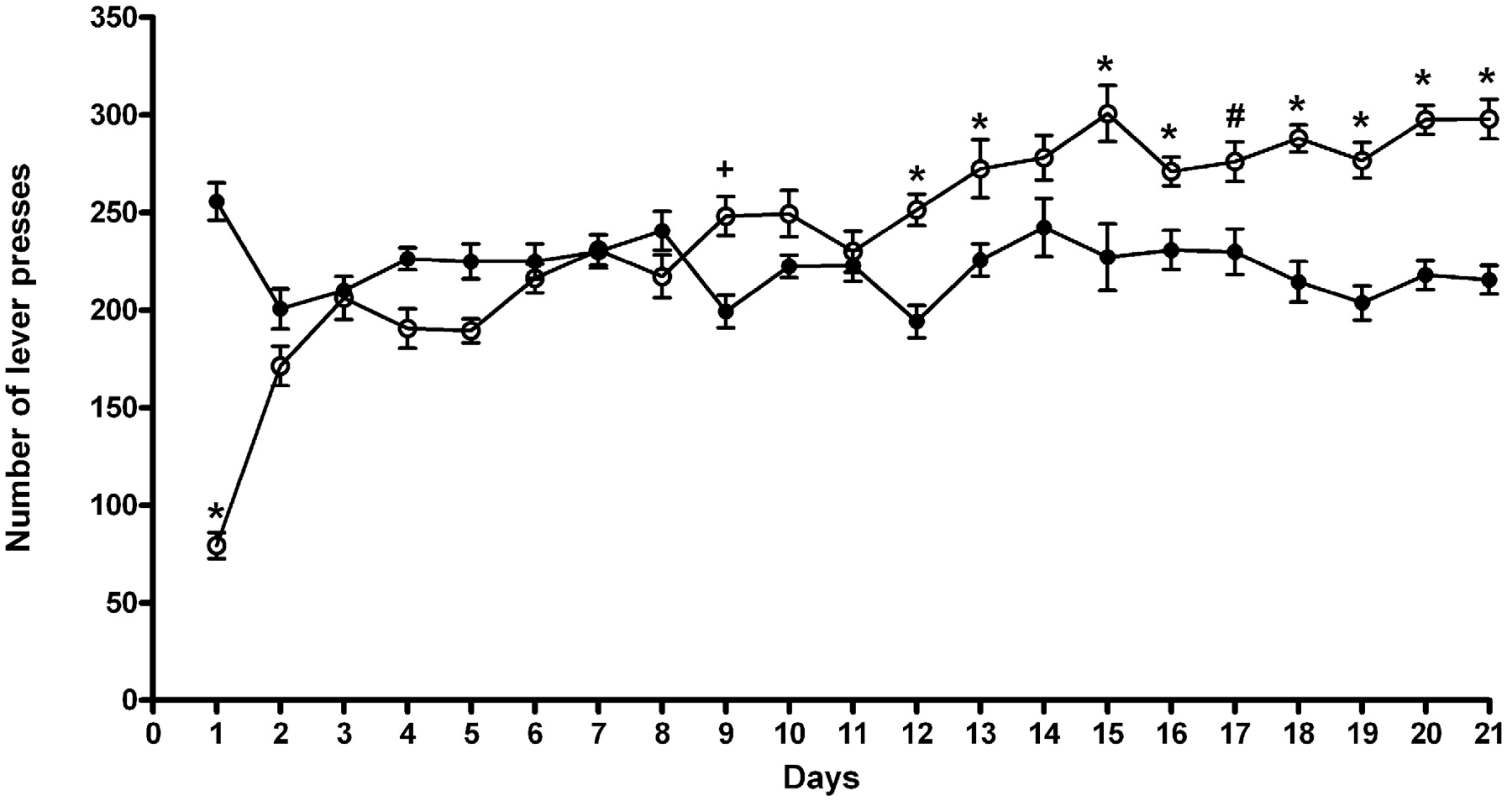
**Number of lever presses during the 21 days of training period.** Each value represents the means ± S.D. of 16 rats. ^#^*p* < 0.05; ^+^*p* < 0.01; ^*^p < 0.001 vs. control groups. (●) CTR, (○) ACD.

The results of a Two-Way ANOVA for repeated measures including ACD treatment as the between-subjects factor and “Days” as within-subjects factor showed a significant effect of time, treatment, and their interaction on the number of responses emitted, *F*_(20, 600)_ = 19.52, *p* < 0.0001; *F*_(1, 30)_ = 5.16, *p* < 0.0304; *F*_(20, 600)_ = 26.81, *p* < 0.0001. Although during the training period both groups showed a similar pattern of operant-drinking behavior, *post-hoc* analysis revealed significant differences along the paradigm (Figure 1). In particular, at the beginning, rats exposed for the first time to ACD in the operant paradigm, showed a lower number of lever presses, and consequently a reduced liquid intake, reaching an amount of ACD consumed of 259 ± 68 mg/kg In the second week of the paradigm, ACD rats' drinking behavior increased, displaying higher number of lever presses and greater liquid intake 3 days of 7, displaying an average intake of 325 ± 21 mg/kg. Values from the third week of training were considered as reference measure of ACD baseline operant behavior. In this week, ACD rats' lever presses were increased significantly with respect to controls, 6 days of 7 (*t* = 7.32, *p* < 0.001; *t* = 3.106, *p* < 0.05; *t* = 6.359, *p* < 0.001; *t* = 6.906, *p* < 0.001; *t* = 5.843, *p* < 0.001; *t* = 5.745, *p* < 0.001), for a mean ACD intake of 355 ± 17 mg/kg. When water intake was measured during the free-drinking hour at the end of the experimental sessions, no differences were observed between the two experimental groups.

#### Extinction

**Figure 2:**
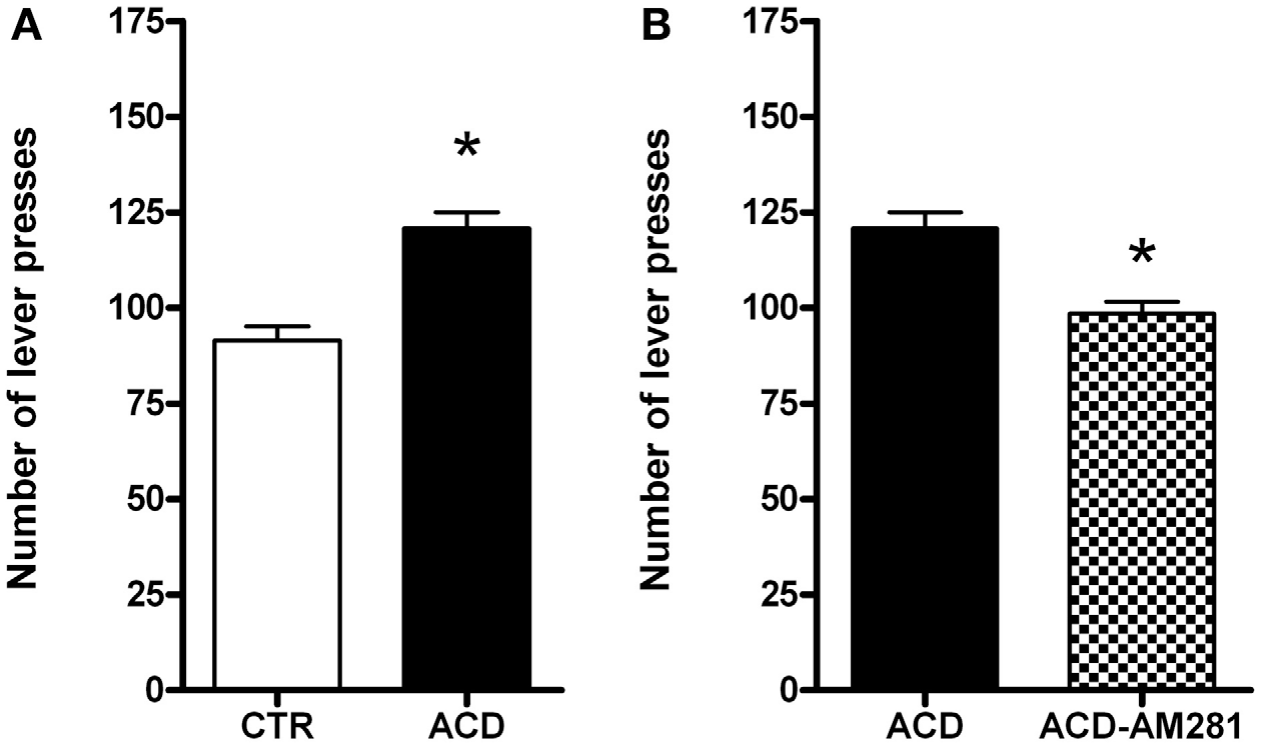
**Effects of ACD (A) and AM281 treatment (B) on the number of lever presses during the extinction day.** Each value represents the means ± S.D. of 16 rats. ^*^p < 0.001 vs. respective control groups.

Rats were tested on the operant condition paradigm to assess drug seeking when reward delivery was suspended. The effects of AM281 treatment on ACD seeking behavior in terms of lever presses were analyzed by a two-tailed Student' *t*-test for unpaired measures. Our data indicated that ACD was able to induce a significant increase in the number of lever presses (*t* = 5.152, *df* = 30, *p* < 0.001) compared to control rats. AM281 administration induced a reduction in the number of lever presses (*t* = 4.196, *df* = 30, *p* < 0.001) in ACD group (Figures 2A,B). AM281 was ineffective on control rats' operant behavior for water.

#### Relapse

**Figure 3:**
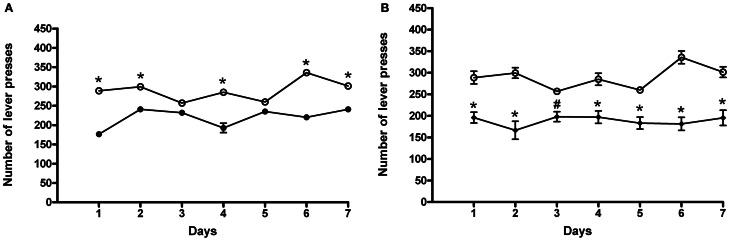
**Effects of ACD (A) and AM281 treatment (B) on the number of lever presses during the relapse periods.** Each value represents the means ± S.D. of 16 rats. ^#^*p* < 0.05; ^*^p < 0.001 vs. respective controls. (●) CTR, (○) ACD, (♦) (ACD-AM281).

Following 7 days of abstinence from ACD self-administration, rats were tested again in the operant chamber to assess whether deprivation could influence their drinking behavior. The results of a Two-Way ANOVA for repeated measures including ACD treatment as the between-subjects factor and “Days” as within-subjects factor showed a significant effect of time, treatment, and their interaction on the number of responses emitted, *F*_(6, 180)_ = 14.21, *p* < 0.0001; *F*_(1, 180)_ = 41.14, *p* < 0.001; *F*_(6, 180)_ = 15.79, *p* < 0.0001. Bonferroni *post-hoc* analysis showed that ACD group displayed an increased number of lever presses on day 1, 2, 4, 6, and 7 (*t* = 8.008., *p* < 0.001; *t* = 4.173, *p* < 0.001; *t* = 6.574, *p* < 0.001; *t* = 8.231, *p* < 0.001; *t* = 4.320, *p* < 0.001) when compared to controls (Figure 3A), reaching an average intake of 409 ± 37 mg/kg. Furthermore, when animals received the selective cannabinoid antagonist AM281, statistical analysis performed by a Two-Way ANOVA for repeated measures, including AM281 treatment as the between-subjects factor and “Days” as within-subjects factor, showed a significant effect of time, treatment, and their interaction on the number of responses emitted, *F*_(6, 180)_ = 4.61 *p* < 0.0002; *F*_(1, 180)_ = 39.57, *p* < 0.001; *F*_(6, 180)_ = 7.82, *p* < 0.0001. Bonferroni *post-hoc* analysis showed that AM281 was able to induce a reduction in the number of lever presses in all days of relapse (*t* = 4.763, *p* < 0.001; *t* = 6.836, *p* < 0.001; *t* = 3.028, *p* < 0.05; *t* = 4.522, *p* < 0.001; *t* = 3.944, *p* < 0.001; *t* = 7.936, *p* < 0.001; *t* = 5.448, *p* < 0.001) in ACD group (Figure 3B) leading to a decrease in the amount of ACD consumed (266 ± 16 mg/kg). No significant differences in the number of lever presses were recorded when AM281 was administered to control rats.

#### Conflict

**Figure 4:**
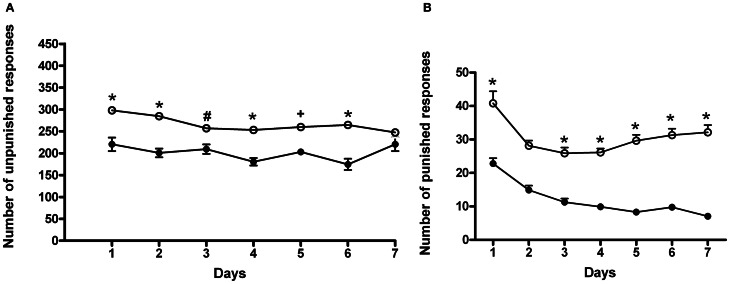
**Differences in the number of unpunished (A) and punished (B) responses during the conflict periods in ACD-and in water-drinking rats.** Each value represents the means ± S.D. of 16 rats. ^#^*p* < 0.05; ^+^*p* < 0.01; ^*^p < 0.001 vs. control groups. (●) CTR, (○) ACD.

In this set of experiments, rats underwent a cyclic schedule of unpunished and punished rewarded responses. Results of a Two-Way ANOVA for repeated measures, including ACD treatment as the between-subjects factor and “Days” as within-subjects factor on unpunished responses, showed a significant effect of time, treatment, and their interaction on the number of responses emitted, *F*_(6, 180)_ = 3.80, *p* < 0.0014; *F*_(1, 180)_ = 74.32, *p* < 0.0001; *F*_(6, 180)_ = 2.35, *p* < 0.0328. Bonferroni *post-hoc* analysis showed that ACD group displayed an increase in the number of unpunished lever presses on day 1, 2, 3, 4, 5, and 6 (*t* = 5.009, *p* < 0.001; *t* = 5.417, *p* < 0.001; *t* = 3.064, *p* < 0.05; *t* = 4.686, *p* < 0.001; *t* = 3.677, *p* < 0.01; *t* = 5.817, *p* < 0.001) when compared to controls (Figure 4A), and greater amount of ACD consumed (377 ± 31 mg/kg). When the parameter “punished responses” was analyzed, a Two-Way ANOVA for repeated measures including ACD treatment as the between-subjects factor and “Days” as within-subjects factor, showed a significant effect of time, treatment, and their interaction on the number of responses emitted, *F*_(6, 180)_ = 16.62, *p* < 0.0001; *F*_(1, 180)_ = 585.43, *p* < 0.0001; *F*_(6, 180)_ = 3.07, *p* < 0.0070. Bonferroni *post-hoc* analysis showed that ACD group displayed an increase in the number of punished lever presses along the conflict period (*t* = 7.584, *p* < 0.001; *t* = 5.583, *p* < 0.001; *t* = 6.162, *p* < 0.001; *t* = 6.847, *p* < 0.001; *t* = 8.980, *p* < 0.001; *t* = 9.085, *p* < 0.001; *t* = 10.56, *p* < 0.001) when compared to controls (Figure 4B), reaching an average amount of ACD ingested of 43 ± 23 mg/kg.

**Figure 5:**
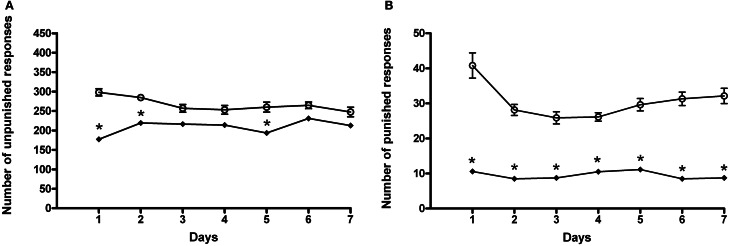
**Effects of the administration of AM281 on the number of unpunished (A) and punished (B) responses during the conflict periods in ACD treated rats.** Each value represents the means ± S.D. of sixteen rats. ^*^p < 0.001 vs. control groups. (○) ACD, (♦) (ACD-AM281).

When animals received AM281, statistical analysis by a Two-Way ANOVA for repeated measures including AM281 treatment as the between-subjects factor and “Days” as within-subjects factor, showed a significant effect of time, treatment, and their interaction on the number of unpunished responses emitted, *F*_(6, 180)_ = 32.43, *p* < 0.0001; *F*_(1, 180)_ = 38.50, *p* < 0.0001; *F*_(6, 180)_ = 5.09, *p* < 0.0001. Bonferroni *post-hoc* analysis showed that AM281 was able to induce a decrease in the number of unpunished lever presses in day 1, 2, 5 (*t* = 7.649, *p* < 0.001; *t* = 4.146, *p* < 0.001; *t* = 4.229, *p* < 0.001) in ACD group compared to respective controls (Figure 5A), and lower amount of ACD consumed (259 ± 21 mg/kg). When the parameter “punished responses” was analyzed after AM281 administration, the results of a Two-Way ANOVA for repeated measures, including ACD treatment as the between-subjects factor and “Days” as within-subjects factor, showed a significant effect of time, treatment, and their interaction on the number of responses emitted, *F*_(6, 180)_ = 5.21, *p* < 0.0001; *F*_(1, 180)_ = 328.73, *p* < 0.0001; *F*_(6, 180)_ = 4.19, *p* < 0.0001. Bonferroni *post-hoc* analysis showed that AM281 was able to induce a decrease in the number of punished lever presses along the conflict period (*t* = 12.01, *p* < 0.001; *t* = 7.791, *p* < 0.001; *t* = 6.779, *p* < 0.001; *t* = 6.203, *p* < 0.001; *t* = 7.345, *p* < 0.001; *t* = 9.057, *p* < 0.001; *t* = 9.280, *p* < 0.001) (Figure 5B) compared to their respective controls, reaching an average amount of ACD ingested of 12 ± 1 mg/kg. No significant difference in the number of unpunished and punished responses was recorded when AM281 was administered to control rats.

## Discussion

The aim of the current study was to evaluate whether ACD could induce and maintain a self-administration drinking behavior in an operant-conditioning paradigm which consisted of training-, extinction-, reinstatement and conflict phases (Cannizzaro et al., 2011; Cacace et al., 2012), in order to demonstrate ACD reinforcing and motivational properties. Afterwards we pointed at exploring the effect of AM281, a CB1 antagonist, on drug-seeking, drug-taking and drug-induced compulsive-like behavior.

There is increasing interest in developing animal models that more closely mimic addiction diagnostic criteria (DSM-IV-TR, American Psychiatric Association, 2000) than classical reinstatement models (Vanderschuren and Ahmed, 2012). Based on this awareness, the experimental protocol implemented in the present study aimed at evaluating the co-occurrence of various aspects of the addictive phenotype, such as the increase in drug use over time; difficulty in restricting drug intake or consuming more than intended; perseveration in drug abuse despite its negative consequences. Our data show that ACD exerted motivational and reinforcing activity, since it was able to induce and maintain an operant drinking behavior; it induced drug-seeking during extinction, and a relapse behavior after 1-week forced abstinence; remarkably, ACD rats displayed a higher emission of punished responses with respect to controls, in a modified Geller–Seifter conflict procedure, which may efficaciously model compulsive drug taking despite negative consequences.

In detail, our results confirm data from a previous study (Cacace et al., 2012), showing that rats readily acquire ACD operant self-administration, according to a fixed ratio of reinforcement, during the training sessions. The number of lever presses for ACD increased over the 3 weeks of training, significantly overcoming control group's lever presses for water from the second week. Moreover when animals were allowed to freely access to water, no significant differences in water intake were observed between the two groups, a finding that accounts for ACD specific motivational effects.

Given that escalation of drug intake is a well-known phenomenon in oral ethanol self-administration studies (Wise, 1973), our data clearly show that ACD shares this feature with ethanol and suggest a direct role in the progressive loss of control over drinking behavior. Difficulty in abstaining from drug use can be studied in animals by assessing drug seeking when the drug is no longer available that is to say extinction paradigm (Ahmed, 2012). Our results indicate that ACD induced a drug-seeking behavior, since ACD group emitted a significantly higher number of lever presses in the extinction experiment with respect to controls. ACD-self administering rats persisted in responding in an attempt to earn the rewarding substance, due to the motivational property of ACD; in fact water self-administering rats showed an earlier extinction of responding, due to the lower value of the reward. This finding allows us to speculate that in ACD group the formation of specific ACD-related associations becomes overly salient, thus enhancing craving for ACD. In the operant conditioning paradigm, reinstatement refers to the rapid resumption of drug-reinforced operant response in animals previously extinguished from drug self-administration training (Marchant et al., 2013). This experimental model mirrors the relapse behavior observed in humans, the most troublesome facet of addiction. ACD oral self-administration sustained a reinstatement in animals. Indeed, ACD-rats, previously exposed to extinction and 1-week forced abstinence, displayed a higher number of lever presses with respect to controls, during the relapse phase. Furthermore, in the first relapse day, lever presses for ACD were higher than in the last training day, suggesting that ACD maintained acute reinforcing strength and motivation even after extinction and deprivation (Martin-Fardon and Weiss, 2013). It is well-known that repeated drug use leads enhances the salience attributed to drugs and drug-associated contexts (Robinson and Berridge, 2008), increasing their consumption following periods of abstinence (Hölter et al., 1998; Rodd et al., 2003). Accordingly, ACD-drinking rats showed a ready resumption of the operant drinking behavior, and displayed a significant effect of deprivation, suggesting that ACD might be also involved in alcohol deprivation effect during relapse (Spanagel and Hölter, 1999).

Drug self-administration in the presence of response-contingent shock punishment highlights the motivational properties of substances and it reliably models compulsive drug use despite adverse consequences (Deroche-Gamonet et al., 2004). Moreover, increasing attention is paid to addiction models that focus on punishment-resistance, as a core feature in capturing the addictive phenotype (Vanderschuren and Ahmed, 2012). Given these premises, we developed the operant-conflict procedure in order to assess whether ACD consumption itself was resistant to aversive consequences associated to drug intake. In the operant-conflict procedure, responses were alternatively paired to a footshock, signaled by a light-cue. In the conflict paradigm rats suffer from the “conflict” between the drive to drink and the fear of the shock: this usually leads to a suppression of conditioned responses for reinforcement. The aversive stimulus has a general dissuasive effect in the operant behavior for water, as it strongly decreases the number of lever presses in CTR group. Despite its highly negative value, the contingent punishment less effectively inhibited operant responding for ACD, and a higher number of punished responses was observed, with respect to controls. It seemed that ACD shares ethanol anti-conflict properties (Baldwin et al., 1991), but since ACD-rats showed an increase both in punished and unpunished responses, it is reasonable to interpret our data recalling ACD strong motivational properties, rather than an anti-conflict effect. Indeed, ACD appears to be a 1000-fold more potent reinforcement than ethanol in the posterior VTA (Rodd et al., 2005). Besides, ACD involvement in recruiting the neuroendocrine stress system (Cannizzaro et al., 2010; Escrig et al., 2012) may be crucial in the development of negative emotional states, thus leading to the progressive loss of control in drinking behavior and compulsive alcohol intake (Koob, 2013). A major finding of the present study was the pharmacological probing of ACD reinforcing and motivational properties, addressing the AM281 effect in the distinct addiction-related behaviors explored, namely drug-seeking, relapse and punishment resistance. Recent preclinical and clinical data indicate that CB1 receptor antagonists, such as SR141716A (SR, Rimonabant), can reduce self-administration and craving for several commonly addictive drugs (Colombo et al., 1998; De Vries et al., 2001; Navarro et al., 2001; Cohen et al., 2005; Rigotti et al., 2009). CB1 function is required for ethanol-mediated activation of VTA DA neurons (Cheer et al., 2007), supporting the hypothesis that ethanol rewarding properties are due in part to ECs release, which likely exerts reduction of GABA inhibition onto VTA dopamine neurons (Lupica and Riegel, 2005; Barrot et al., 2012). This effect is quite specific, since the neuroanatomical loci of the SR-mediated reduction in ethanol self-administration involve brain regions typically associated with addiction; indeed SR microinjections into VTA, medial prefrontal cortex and NAcc reduce ethanol self-administration, whereas injections into the dorsal striatum do not affect the number of responses for ethanol (Caillé et al., 2007; Hansson et al., 2007; Malinen and Hyytiä, 2008). AM281 is structurally related to SR, but displays higher affinity and specificity for CB1 receptor (Gatley et al., 1998), since it does not interact with GPR55 and opioid receptors (Seely et al., 2012). Our results show that AM281 administration was able to decrease ACD-seeking, ACD-relapse after forced abstinence, and ACD-induced resistance to punishment in highly predictive experimental procedures. Indeed, animals receiving the CB1 antagonist emitted a lower number of responses for ACD with respect to vehicle group during extinction. This evidence suggests that ACD positive incentive properties underlie perseveration in lever pressing when reinforce delivery is suspended. No significant effect was recorded in water-administering rats, ruling out an aspecific action on operant responding. AM281 administration induced a significant reduction in lever-pressing during the relapse session, when compared to their respective controls. This finding further suggests a role for CB1 receptor in ACD-induced as well as it has been reported in alcohol-related addictive behavior (Serrano and Parsons, 2011). Ultimately, AM281 administration in the conflict paradigm decreased the number of punished lever presses for ACD, and a similar though less evident effect was observed in unpunished ones. At this regard, an aspecific effect of the CB1 antagonist on motor activity seems unlikely, since AM281 administration was able to affect unpunished responses for ACD discontinuously, while the reduction of punished responses occurred along the whole conflict period. This effect may be related to a decrease in the incentive for lever-pressing due to the pharmacological treatment. The clear influence of CB1 receptor activity on ACD-induced punishment resistance further highlights the involvement of the reward-processing machinery as the intrinsic mechanisms underlying ACD-related behavioral features. As a matter of fact, these data provide evidence of ACD incentive properties, whose contribution must be taken into account in studying and treating ethanol-related behaviors. The neural substrates underpinning rewarding properties of orally self-administer ACD involve CB1 receptors, which are able to indirectly modulate DA mesocorticolimbic pathway. Drugs able to manipulate EC system might represent a useful therapeutic strategy affecting both ethanol and its neuroactive metabolite actions on crucial addiction-related behaviors, such as drug seeking, relapse and drug abuse despite negative consequences. This study aims at representing a step forward in elucidating the complex framework of actors playing a role in maintaining ethanol addiction; nevertheless further efforts are needed to fully characterize the actual contribution of ACD to ethanol's effects.

### Conflict of interest statement

The authors declare that the research was conducted in the absence of any commercial or financial relationships that could be construed as a potential conflict of interest.
